# Copper-regulated cell death after spinal cord injury: evidence boundaries for non-coding RNA, epigenetics and cuproptosis

**DOI:** 10.3389/fnmol.2026.1885261

**Published:** 2026-09-10

**Authors:** Jun Tao, Guobing Wang, Guoying Jiang, Wenqi Feng

**Affiliations:** 1 Department of Rehabilitation Medicine, Yibin Traditional Chinese Medicine Hospital, Yibin, Sichuan, China; 2 SCATCM Key Laboratory of Traditional Chinese Medicine Health Products Research and Clinical Evaluation (Yibin Traditional Chinese Medicine Hospital), Yibin, Sichuan, China; 3 Yibin Clinical Research Center for Traditional Chinese Medicine Health Products, Yibin, Sichuan, China; 4 Department of Pathology, Yibin Traditional Chinese Medicine Hospital, Yibin, Sichuan, China

**Keywords:** copper homeostasis, cuproptosis, epigenetics, molecular neurobiology, non-coding RNA, secondary injury, spinal cord injury

## Abstract

**Background:**

Spinal cord injury (SCI) initiates a secondary-injury cascade involving mitochondrial dysfunction, metal dyshomeostasis, neuroinflammation, and regulated cell death. Cuproptosis, a copper-dependent form of regulated cell death driven by lipoylated mitochondrial protein aggregation, has reframed copper dyshomeostasis as a potential molecular vulnerability rather than a passive trace-metal disturbance.

**Main body:**

This targeted narrative review critically synthesizes the FDX1–lipoylation–DLAT/DLST cuproptosis pathway, copper trafficking and redistribution after SCI, and the evidence boundary between spinal cord ischemia–reperfusion injury (SCIRI) and traumatic SCI. We evaluate two distinct but interacting regulatory layers: non-coding RNAs (ncRNAs), including microRNAs, long non-coding RNAs, circular RNAs, and extracellular-vesicle RNAs; and epigenetic regulation, including DNA methylation, histone modifications, and RNA modifications. Evidence is graded to distinguish established canonical biochemistry, the strongest direct preclinical mechanistic evidence in spinal-cord models, associative or hypothesis-generating traumatic-SCI evidence, non-SCI mechanistic validation, and testable hypotheses.

**Conclusion:**

At present the ncRNA–epigenetics–cuproptosis axis represents a molecular-neurobiological framework that still requires experimental validation rather than a clinically actionable pathway. The highest-priority next step is temporally and spatially resolved traumatic SCI validation that combines copper mapping, cell-type-specific cuproptosis execution assays, immune profiling, and genetic or pharmacological rescue experiments.

## Introduction: the unresolved secondary-injury window in SCI

### Clinical burden and the limits of current treatment

The Global Burden of Disease 2021 analysis estimated 574,502 incident SCI cases worldwide [95% uncertainty interval (UI), 440,219–757,445] and 15,400,682 prevalent cases (95% UI, 14,009,114–17,075,106), indicating that SCI is a major source of long-term neurological disability (Lu et al., 2025). Traumatic SCI initiates mechanical tissue disruption, hemorrhage, ischemia, excitotoxicity, inflammation, oxidative stress, and multiple regulated cell-death programs that evolve over hours to weeks, creating a biologically plausible but clinically difficult therapeutic window (Ahuja et al., 2017; Alizadeh et al., 2019).

The translational problem is not merely a lack of candidate pathways. Many interventions reduce lesion volume or improve locomotor scores in rodents but have not become broadly accepted disease-modifying therapies in humans (Ahuja et al., 2017). Methylprednisolone sodium succinate (MPSS) illustrates this gap: systematic review evidence supports at most time-window-dependent and guideline-conditional use, while safety concerns and modest effect sizes prevent it from being considered a robust disease-modifying therapy (Fehlings et al., 2017).

### Cuproptosis as a candidate, not yet established, SCI mechanism

Cell death after SCI is now better viewed as a network of interacting death modules rather than a linear apoptosis cascade. Ferroptosis, pyroptosis, necroptosis, autophagy-dependent death, and apoptosis have distinct molecular initiators, yet they converge on mitochondrial dysfunction, redox imbalance, damage-associated molecular pattern (DAMP) release, and inflammatory amplification (Galluzzi et al., 2018; Tran et al., 2018).

Cuproptosis is attractive in this network because it links metal dyshomeostasis to mitochondrial metabolism. Tsvetkov and colleagues showed that copper can induce death by binding lipoylated tricarboxylic acid (TCA) cycle proteins, promoting protein aggregation and iron–sulfur (Fe-S) cluster protein loss; this establishes the biochemical mechanism but does not, by itself, prove occurrence in traumatic SCI (Tsvetkov et al., 2022; Tang et al., 2022).

Recent work has connected copper dyshomeostasis to broader CNS disease contexts, and a SCIRI study has provided the most complete spinal cord evidence for ATP7B downregulation, copper accumulation, DLAT aggregation, Fe-S protein loss, and chelation-sensitive neuronal injury (Zhu et al., 2024; Xie et al., 2025). Reviews of copper biology in SCI support biological plausibility, but they also highlight that direct spatiotemporal copper mapping in traumatic contusion, compression, and laceration models remains scarce (Ni et al., 2026).

### The core argument of this review

This review is organized around one central question: does the ncRNA–epigenetics–cuproptosis axis represent a causal and targetable driver of SCI secondary injury, or is it currently an emerging but under-validated concept in molecular neuroscience? We argue that an intermediate position is most reasonable. Cuproptosis chemistry is established; SCIRI currently provides the strongest direct preclinical mechanistic evidence in a spinal-cord model; traumatic-SCI CRG studies remain associative or hypothesis-generating; and ncRNA or epigenetic regulation of cuproptosis in spinal tissue remains largely unvalidated.

The review therefore avoids a simple mechanism–drug–clinical sequence. Each section sets out what is established, what is uncertain, and what would be needed to raise the claim to a higher evidence level. Table 1 summarizes the main evidence boundaries and is intended to limit overinterpretation of bioinformatic or cross-disease findings. The categories are an author-proposed descriptive framework, not a formal GRADE assessment.

**TABLE 1:** Evidence map for cuproptosis-related claims in spinal cord injury.

Claim	Most direct supporting evidence	Evidence grade used in this review	Key limitation	Recommended wording
Canonical cuproptosis is a copper-dependent mitochondrial death mechanism	Mechanistic cell studies showing lipoylated TCA-cycle protein aggregation and Fe-S protein loss (Tsvetkov et al., 2022; Tang et al., 2022)	Established canonical biochemical evidence	Not disease- or tissue-specific	Established mechanism; disease relevance requires contextual validation
SCIRI can trigger neuronal cuproptosis	ATP7B–FDX1–DLAT chain with copper accumulation and TM rescue (Xie et al., 2025)	Strongest direct preclinical mechanistic evidence in a spinal-cord model	Single SCIRI model; limited temporal, sex, and cell-type resolution	Direct preclinical mechanistic evidence in SCIRI; not equivalent to traumatic-SCI or human evidence
Traumatic SCI is associated with cuproptosis-related gene signatures	CRG bioinformatics and emerging SCI studies (Li et al., 2023; Mao et al., 2025; Zhou et al., 2025)	Associative/hypothesis-generating evidence	No definitive traumatic-tissue copper mapping, aggregation, or copper-specific rescue	Association only; cuproptosis execution remains unproven
ncRNAs may regulate cuproptosis susceptibility in SCI	SCI ncRNA biology and EV studies (Nieto-Diaz et al., 2014; Shi et al., 2018; Li et al., 2018), plus non-SCI cuproptosis regulation (El-Ashmawy et al., 2025; Du et al., 2025)	Indirect mechanistic plausibility for SCI	No validated SCI ncRNA–CRG interaction with cuproptosis rescue	Candidate regulatory layer requiring causal validation
Epigenetic states may tune cuproptosis thresholds in SCI	SCI epigenetic and RNA-modification evidence (Wahane et al., 2019; Yu et al., 2022; Guo et al., 2023)	Indirect mechanistic plausibility for SCI	Few cell-resolved CRG promoter/enhancer or RNA-modification studies	Testable upstream control mechanism

Recent SCI-focused reviews have underscored cuproptosis as an emerging mechanism in secondary injury and immune dysregulation; the present review adds a stratified framework that separates canonical biochemistry, SCIRI proof, traumatic SCI association, and ncRNA/epigenetic hypotheses before therapeutic translation (Li et al., 2026).

## Copper biology in the injured spinal cord

### Normal copper trafficking: a low-free-copper system

Copper is essential for mitochondrial respiration, antioxidant defense, and neurotransmitter metabolism, yet unbuffered copper is toxic; intracellular free copper is therefore maintained at extremely low levels by transporters and chaperones rather than allowed to diffuse freely (Lutsenko et al., 2007; Baker et al., 2017).

SLC31A1/CTR1 mediates high-affinity Cu(I) uptake, ATOX1 delivers copper to ATP7A/B in the secretory pathway, CCS transfers copper to SOD1, and COX17 contributes to cytochrome c oxidase assembly in mitochondria (Lutsenko et al., 2007; Baker et al., 2017). ATP7A and ATP7B both load copper onto cuproenzymes and can redistribute toward membranes during copper excess, making them key nodes for both physiology and toxicity (Lutsenko et al., 2007).

### Why SCI may perturb copper without proving overload

SCI creates several conditions compatible with local copper dysregulation: hemorrhage introduces blood-derived metal pools, blood–spinal cord barrier (BSCB) breakdown changes vascular permeability, damaged mitochondria can redistribute intracellular copper, and infiltrating immune cells may alter metal availability in the lesion environment (Ahuja et al., 2017; Ni et al., 2026). These processes make copper dyshomeostasis plausible, but traumatic SCI cannot yet be characterized as a copper-overload disease until direct tissue mapping supports that claim.

Excess copper can participate in redox cycling, interact with thiol-rich proteins, destabilize Fe-S cluster proteins, and overlap with ferroptosis-like redox injury; these mechanisms are supported by general copper toxicology and cuproptosis literature rather than by traumatic SCI-specific proof (Gaetke et al., 2014; Chen et al., 2022). This distinction matters because a biologically plausible metal response does not automatically identify the dominant cell death mechanism in a complex lesion.

## Molecular execution of cuproptosis

### The FDX1–lipoylation–DLAT axis

Canonical cuproptosis requires mitochondrial metabolism and protein lipoylation. FDX1 functions as an upstream regulator in copper-induced death models, supporting copper redox conversion and lipoylation-dependent vulnerability; lipoylated DLAT and DLST are principal mitochondrial targets (Tsvetkov et al., 2022; Rowland et al., 2018).

Elesclomol–copper studies and FDX1 mutational analyses refine this model by showing that copper delivery, FDX1-dependent redox chemistry, and structural determinants of FDX1 influence cuproptosis sensitivity (Zulkifli et al., 2023; Hsiao et al., 2025). These studies strengthen the biochemical framework but were performed largely outside SCI models, and their relevance to SCI is therefore mechanistic rather than confirmatory of disease occurrence.

### Detection standards for SCI studies

A recurrent weakness in cuproptosis papers is reliance on cuproptosis-related gene (CRG) expression scores alone. For SCI, a more convincing author-suggested working standard would include direct copper measurement or imaging; transporter changes such as ATP7B or SLC31A1 dysregulation; FDX1, LIAS, LIPT1/2, DLAT, and DLST assessment at RNA and protein levels; evidence of lipoylated protein aggregation; Fe-S cluster protein loss; and rescue by copper chelation or genetic perturbation (Tsvetkov et al., 2022; Xie et al., 2025).

Under this author-proposed working framework, studies meeting only one or two criteria should be described as hypothesis-generating. This threshold is intended to discipline interpretation rather than to represent an established consensus standard. In the absence of aggregation assays and rescue experiments, changes in CRG expression may reflect mitochondrial stress, inflammatory-cell admixture, or altered cell composition rather than cuproptosis execution.

## Current evidence for cuproptosis in spinal cord injury

### Bioinformatic evidence: useful but not causal

Bioinformatic analyses of SCI transcriptomic datasets have identified associations between CRGs, immune infiltration signatures, and candidate biomarkers, including DLD-mediated models, Mpeg1 hub-gene analysis, and machine-learning panels involving SLC31A1, DBT, DLST, and LIAS (Li et al., 2023; Mao et al., 2025; Zhou et al., 2025). These studies are valuable for prioritizing targets, but bulk transcriptomics cannot distinguish changes in injured neurons from shifts in immune-cell composition.

Their limitations are consistent: small public datasets, heterogeneous injury models and time points, limited external validation, no direct copper measurement, and little protein-level confirmation. These studies should therefore be cited as hypothesis-generating work rather than as proof that traumatic SCI undergoes cuproptosis.

### Direct mechanistic evidence: strongest direct preclinical evidence in SCIRI

The strongest direct preclinical spinal-cord evidence currently comes from SCIRI. Xie et al. reported ATP7B downregulation, intracellular copper accumulation, FDX1 activation, DLAT lipoylation and aggregation, Fe-S cluster protein degradation, neuronal death, and functional rescue with tetrathiomolybdate (TM) (Xie et al., 2025). This chain satisfies most proposed evidence elements for cuproptosis and is best treated as a provisional mechanistic anchor rather than definitive evidence for traumatic SCI or human disease.

The same study also defines the boundary of inference. SCIRI differs from traumatic SCI in the timing of oxidative bursts, vascular pathology, cellular necrosis, inflammatory recruitment, and mechanical tissue disruption. Therefore, extension from SCIRI to contusion, compression, or laceration models should be framed as a priority hypothesis rather than an established conclusion (Xie et al., 2025; Xu et al., 2025).

### Evidence map

Table 1 summarizes the descriptive evidence framework used throughout this review: canonical cuproptosis biochemistry is established; SCIRI provides the strongest direct preclinical mechanistic evidence in a spinal-cord model; traumatic SCI has associative or hypothesis-generating evidence; and ncRNA/epigenetic regulation remains largely extrapolated. The review-level SANRA self-appraisal and study-level reporting/validation features are provided in Supplementary Tables 3, 4.

The evidence grade reflects directness and mechanistic depth, not a pooled risk-of-bias score. Detailed reporting appraisal is provided in Supplementary material.

## Crosstalk with other regulated cell death programs

### Cuproptosis and ferroptosis: shared metal stress, different execution logic

Cuproptosis and ferroptosis intersect through metal toxicity, mitochondrial injury, reactive oxygen species (ROS) generation, and Fe-S cluster instability, but their execution logic differs. Ferroptosis is defined by iron-dependent lipid peroxidation and GPX4-linked antioxidant failure, whereas cuproptosis is centered on copper binding to lipoylated mitochondrial proteins and proteotoxic aggregation (Galluzzi et al., 2018; Tsvetkov et al., 2022).

The difference has practical consequences. A rise in ROS or lipid peroxidation after SCI does not prove cuproptosis, and copper accumulation does not prove ferroptosis. Primary traumatic-SCI studies have shown that deferoxamine, the ferroptosis inhibitor SRS 16-86, and liproxstatin-1 can modify lipid-peroxidation/GPX4-linked endpoints and improve selected functional outcomes (Yao et al., 2019; Zhang et al., 2019; Fan et al., 2021). A crosstalk experiment should therefore measure lipid peroxidation, the GPX4/ACSL4 axis, copper distribution, lipoylated DLAT/DLST aggregation, and Fe-S protein loss in the same samples, with pathway-specific rescue.

### Cuproptosis, pyroptosis, necroptosis, and autophagy: hypotheses requiring model-specific testing

Copper can regulate the canonical NLRP3 inflammasome in selected experimental systems, and NLRP3 is implicated in SCI neuroinflammation; however, the direction and magnitude of copper-driven inflammasome activation in SCI remain context-dependent (Deigendesch et al., 2018; Jiang et al., 2017).

**FIGURE 1:**
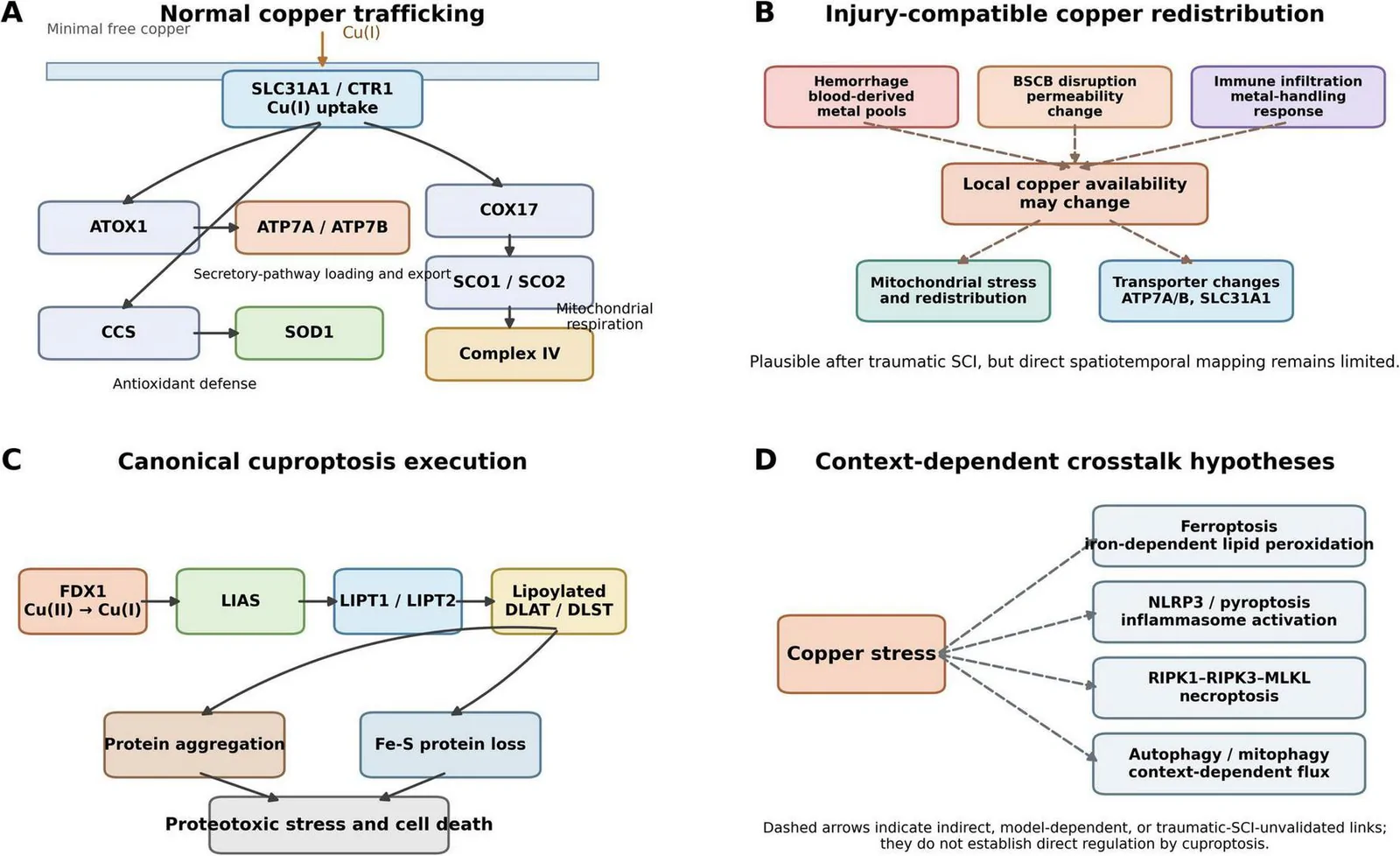
Copper dyshomeostasis and cuproptosis in the injured spinal cord. **(A)** Separates the normal copper-trafficking routes SLC31A1/CTR1→ATOX1→ATP7A/B, CCS→SOD1, and COX17→SCO1/SCO2→complex IV. **(B)** Summarizes injury-compatible but incompletely mapped sources of copper redistribution. **(C)** Shows canonical FDX1–lipoylation–DLAT/DLST execution. In panel **(D)**, each dashed arrow from copper stress denotes a proposed, context-dependent interface with iron-dependent lipid peroxidation/ferroptosis, NLRP3 inflammasome activation/pyroptosis, RIPK1–RIPK3–MLKL necroptosis, or autophagy/mitophagy. Solid arrows in panels **(A,C)** denote canonical mechanisms; dashed arrows denote indirect, model-dependent, or traumatic-SCI-unvalidated links and do not establish direct regulation by cuproptosis.

Necroptosis and autophagy are also relevant to SCI, but linking them to cuproptosis requires copper-specific perturbation. Necroptosis inhibition can protect tissue in traumatic-SCI models, and autophagy can be protective or harmful depending on timing and cellular context (Wang et al., 2014; Lipinski et al., 2015). These primary SCI observations establish the individual pathways, not direct cuproptosis crosstalk. Figure 1 therefore marks all crosstalk edges as indirect or model-dependent.

## Non-coding RNA regulation of cuproptosis after SCI

### miRNAs: strongest in SCI biology, weakest in SCI-cuproptosis causality

miRNAs regulate inflammation, apoptosis, axonal growth, and scar-associated pathways after SCI, giving them strong disease-context relevance (Nieto-Diaz et al., 2014). The weak link is specificity: predicted miRNA targeting of CRGs such as SLC31A1, ATP7B, FDX1, DLAT, or LIAS generally comes from databases or non-SCI studies, not from spinal tissue luciferase validation, AGO2-CLIP, or cell-type-specific gain/loss-of-function assays.

Authors should therefore avoid claiming that a given miRNA controls SCI cuproptosis unless the study demonstrates direct binding, target modulation, cuproptosis phenotypes, and rescue. The more accurate formulation is that miRNAs provide a tractable candidate layer for tuning copper transporter and CRG expression.

### lncRNAs, circRNAs, and extracellular vesicle (EV) RNAs

lncRNAs can regulate gene expression through chromatin scaffolding, transcriptional control, and ceRNA networks; SCI-associated lncRNAs have been linked to inflammation and cell death, but direct intersection with cuproptosis remains largely absent (Statello et al., 2021; Shi et al., 2018). Cancer-focused reviews on lncRNA- or ncRNA-mediated cuproptosis are useful for mechanism discovery, but they cannot be directly transferred to neurons, oligodendrocytes, or microglia without experimental testing (El-Ashmawy et al., 2025; Du et al., 2025).

circRNAs are stable, tissue-enriched RNA molecules that can function as miRNA sponges, and EVs provide a plausible route for intercellular RNA communication in the injured spinal cord (Kristensen et al., 2019; Panda, 2018; Pegtel and Gould, 2019). Exosome studies in SCI demonstrate that RNA-modified vesicles can affect recovery, but they have not yet established propagation or suppression of cuproptosis sensitivity (Li et al., 2018).

### Evidence tiers for ncRNA–cuproptosis interactions

Table 2 provides a stricter interpretation framework. The main conclusion is cautionary but important: there is currently no Tier 1 evidence for a directly validated ncRNA–cuproptosis axis in SCI.

**TABLE 2:** Evidence stratification for ncRNA–cuproptosis regulation in SCI.

Evidence tier	Definition	Current SCI status	Examples or candidate evidence	Actionable next step
Tier 1	Directly validated ncRNA–CRG/cuproptosis interaction in SCI	Not yet available	No SCI study currently demonstrates ncRNA binding plus cuproptosis rescue	Prioritize direct binding, cell-type-restricted perturbation, and rescue in neurons, oligodendrocytes, microglia/macrophages, astrocytes, and vascular cells
Tier 2	Directly validated in non-SCI models	Indirect relevance only	Cancer and other non-SCI ncRNA–cuproptosis literature (El-Ashmawy et al., 2025; Du et al., 2025)	Replicate only mechanistically strong candidates in SCI-relevant cell systems
Tier 3	Bioinformatic prediction in SCI datasets	Hypothesis-generating	CRG-based immune and machine-learning studies (Li et al., 2023; Mao et al., 2025; Zhou et al., 2025)	Validate at protein level and resolve cell-type origin
Tier 4	Mechanistic plausibility without direct validation	Common in current narratives	EV RNA transfer or ceRNA modulation of CRG expression; Li et al. (2018) supports EV-miRNA benefit in SCI recovery but not CRG/cuproptosis regulation (Pegtel and Gould, 2019; Li et al., 2018)	Label explicitly as hypothesis and avoid therapeutic claims

## Epigenetic regulation upstream and downstream of cuproptosis

### DNA methylation and histone modifications

DNA methylation and histone modifications provide durable mechanisms by which injury history could alter cell death thresholds. lncRNAs can recruit chromatin regulators, and SCI studies support broad epigenetic involvement in axon regeneration, glial activation, and injury responses (Mercer and Mattick, 2013; Wahane et al., 2019).

However, the specific claim that ATP7B hypermethylation, SLC31A1 demethylation, or DLAT promoter remodeling controls cuproptosis in SCI is not yet supported by direct spinal cord evidence. It should be presented as a testable hypothesis requiring bisulfite sequencing, CUT&Tag or ChIP-seq, ATAC-seq, and CRG expression validation in the same cell populations.

Epigenetic barriers to axon regeneration have been experimentally demonstrated, and valproic acid has shown preclinical SCI benefit (Weng et al., 2017; Lv et al., 2012). However, valproic acid is pleiotropic and its actions extend beyond histone deacetylase inhibition; the observed benefit therefore cannot be attributed specifically to an HDAC-mediated mechanism. BET inhibition is mechanistically plausible because BET proteins read acetylated chromatin, but direct SCI-cuproptosis evidence is absent (Filippakopoulos et al., 2010). These findings support chromatin plasticity as a modifiable injury response, yet none directly prove cuproptosis modulation in SCI.

### RNA epigenetics

m6A modification can alter mRNA structure, stability, translation, and reader-protein recruitment, making it a plausible regulator of CRG transcripts (He and He, 2021; Liu et al., 2017). SCI studies have mapped m6A changes after traumatic injury, and METTL3 has been implicated in spinal cord neuronal apoptosis through Bcl-2 m6A modification, but these studies focus on apoptosis rather than cuproptosis (Yu et al., 2022; Guo et al., 2023).

A-to-I RNA editing is even less explored in SCI cuproptosis. Its most defensible role at present is as a candidate modifier of miRNA target recognition or CRG transcript fate, a hypothesis grounded in RNA editing biology rather than demonstrated spinal cord evidence (Nishikura, 2016).

### Bidirectional loop between ncRNAs and epigenetics

**FIGURE 2:**
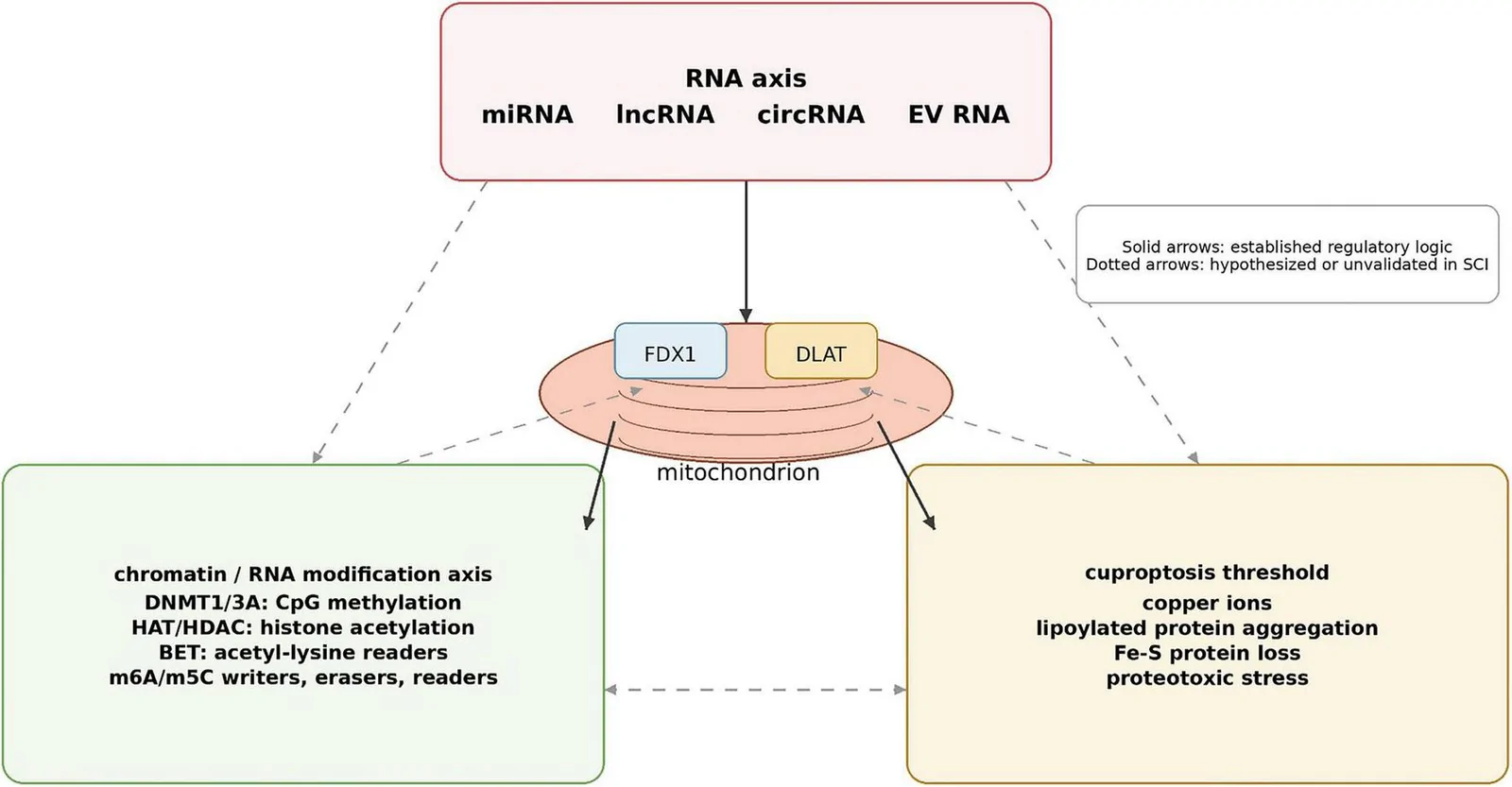
Proposed ncRNA–epigenetics–cuproptosis triaxial regulatory network. The RNA axis includes miRNA, lncRNA, circRNA, and EV RNA; the chromatin/RNA modification axis includes DNMT1/3A-associated CpG methylation, HAT/HDAC-mediated histone acetylation, BET acetyl-lysine readers, and m6A/m5C writer, eraser, and reader proteins. Solid arrows represent established regulatory logic, whereas dotted arrows indicate hypothesized or SCI-unvalidated relationships that require causal testing.

The regulatory architecture is likely bidirectional. ncRNAs can recruit epigenetic enzymes, while epigenetic marks can regulate ncRNA transcription; the miR-29–DNMT axis is a classic non-SCI example of RNA-guided epigenetic feedback (Fabbri et al., 2007). Figure 2 depicts this as a triaxial model in which RNA regulators and chromatin/RNA modifications alter the cuproptosis threshold rather than directly proving cuproptosis execution.

### Key technical bottlenecks and experimental solutions

Cell-type-selective manipulation is a central bottleneck because the injured cord contains neurons, oligodendrocytes, astrocytes, resident microglia, infiltrating macrophages, endothelial cells, and pericytes within the same lesion. AAV capsid, route, dose, and promoter should therefore be treated as empirical variables rather than assumed to be cell-specific. Candidate constructs should be benchmarked with reporter expression, immunophenotyping, and, where feasible, single-cell or spatial readouts; resident microglia should be distinguished from monocyte-derived macrophages. Synthetic-promoter AAV libraries provide a platform-level precedent, but specificity must be re-established in the injured spinal cord (Jüttner et al., 2019).

For ncRNAs, a minimally persuasive design should combine cell-type-restricted gain or loss of function, direct target validation (dual-luciferase plus AGO2-RIP/CLIP where applicable), protein-level CRG measurement, cuproptosis-execution endpoints, and rescue. A change in RNA abundance, a predicted ceRNA network, or an immune correlation should not be treated as causal without this chain of evidence.

For epigenetic hypotheses, locus-specific editing can move the field beyond association. dCas9-p300 can test whether acetylation at a candidate promoter or enhancer is sufficient to activate a CRG, whereas dCas9-DNMT3A (including multivalent recruitment configurations such as SunTag) or dCas9-TET1 can test whether targeted methylation or demethylation changes ATP7B, SLC31A1, FDX1, DLAT, or related genes (Hilton et al., 2015; Liu et al., 2016). Each intervention should be linked to local chromatin readouts, target-gene expression, copper distribution, DLAT/DLST aggregation, Fe-S protein loss, and chelation or genetic rescue. These systems are experimental validation tools rather than near-term SCI therapies.

## Cell-type specificity: where the hypothesis is strongest and weakest

### Neurons and oligodendrocytes

Neurons are plausible cuproptosis targets because they are highly dependent on mitochondrial metabolism, and SCIRI evidence directly implicates neuronal cuproptosis (Xie et al., 2025; Courtine and Sofroniew, 2019). Yet traumatic SCI includes mechanical axotomy, excitotoxicity, necrosis, inflammation, and demyelination, so neuronal cuproptosis should be tested alongside competing death pathways rather than assumed to be dominant.

Oligodendrocytes are also plausible targets because myelination and axonal support impose high metabolic demands, and remyelination failure is a major repair bottleneck after SCI (Bradl and Lassmann, 2010; Plemel et al., 2014). Still, the leap from metabolic vulnerability to cuproptosis susceptibility requires oligodendrocyte-specific copper measurement, DLAT aggregation, Fe-S protein assays, and rescue experiments.

### Microglia, macrophages, astrocytes, and vascular cells

Microglia and infiltrating macrophages are central to SCI neuroinflammation and metal handling, but their cuproptosis role may be dual: copper can support antimicrobial and immune functions while excess copper may promote inflammatory toxicity (Stafford et al., 2013; Orihuela et al., 2016). A simple M1/M2 framework is insufficient because macrophage states after SCI are continuous, context-dependent, and metabolically plastic.

Astrocytes may buffer extracellular stress, shape inflammatory toxicity, or become reactive contributors to secondary damage; reactive astrocyte nomenclature and function are now recognized as context-dependent rather than binary (Liddelow et al., 2017; Escartin et al., 2021). Endothelial cells and pericytes regulate BSCB integrity and therefore may influence copper flux into the lesion, but their cuproptosis susceptibility has not been directly tested in SCI (Bartanusz et al., 2011).

**FIGURE 3:**
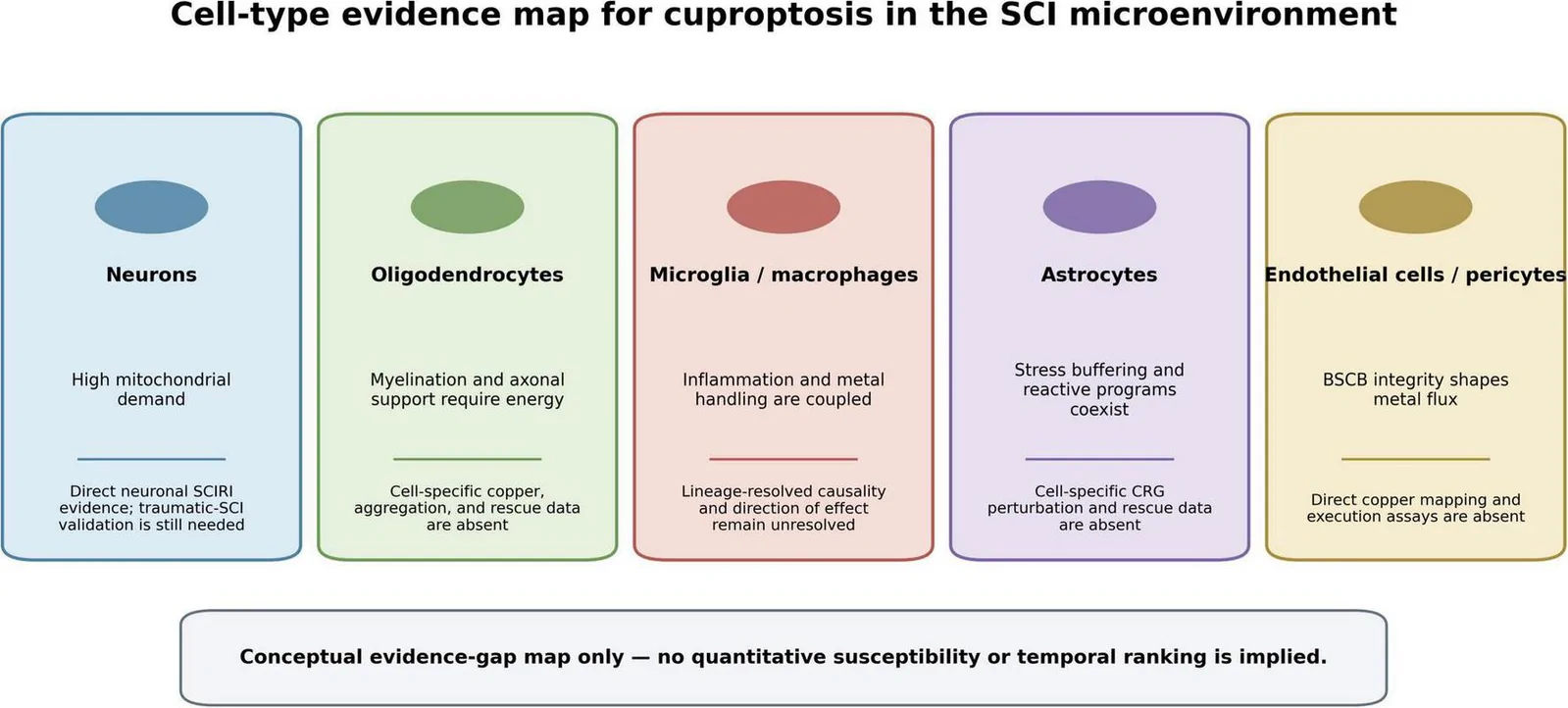
Cell-type evidence map for cuproptosis in the SCI microenvironment. Five candidate populations–neurons, oligodendrocytes, microglia/macrophages, astrocytes, and endothelial cells/pericytes–are presented with equal visual weight. Each panel pairs biological plausibility with the decisive evidence gap. The figure is conceptual only; no quantitative susceptibility or temporal ranking is implied.

Single-cell analysis of injured spinal cord tissue provides a path toward resolving cell-type specificity, but transcriptomic states still require metallomic and protein-level validation before they can be called cuproptotic (Milich et al., 2021). Figure 3 is a conceptual evidence-gap map. It deliberately assigns equal visual weight to five candidate populations and does not imply a quantitative susceptibility or temporal ranking.

## Therapeutic implications: prioritize validation before clinical translation

### Copper chelation and copper redistribution

TM is the most relevant copper-lowering intervention for SCI-cuproptosis because it rescued neuronal injury in SCIRI, but that result is preclinical and model-specific (Xie et al., 2025). Wilson disease experience with copper-lowering therapy provides safety concepts, monitoring strategies, and toxicity concerns, yet it does not establish efficacy in acute SCI (Brewer, 2009; European Association for the Study of the Liver, 2012).

A major risk is overshooting copper reduction. Copper is required for mitochondrial respiration, antioxidant defense, and neural function; chronic or excessive depletion can cause anemia, immune dysfunction, or neurological toxicity, as illustrated by copper-deficiency literature and Wilson disease treatment monitoring (European Association for the Study of the Liver, 2012; Olivares et al., 2000). Any SCI chelation strategy therefore needs a defined time window, lesion-stage stratification, and copper biomarker monitoring rather than continuous non-specific copper suppression.

### RNA and epigenetic interventions

RNA therapeutics, including miRNA mimics, antagomirs, antisense oligonucleotides (ASOs), siRNAs, and CRISPR-Cas13 systems, offer theoretical precision but face delivery, immunogenicity, off-target, and cell-type specificity barriers in CNS trauma (Rupaimoole and Slack, 2017; Abudayyeh et al., 2017). In the absence of Tier 1 SCI evidence, RNA targeting of cuproptosis is best used as a mechanistic validation tool rather than presented as a near-term clinical therapy.

Epigenetic drugs may reshape inflammatory and regenerative programs, but their broad action is both strength and liability. Valproic acid has shown preclinical benefit in SCI models (Lv et al., 2012), and broader CNS-injury evidence supports plausibility (Fessler et al., 2013), yet its pleiotropic actions prevent attribution of benefit to HDAC inhibition alone. Global chromatin modulation may affect multiple cell types and pathways unrelated to copper-dependent death.

### Delivery platforms and combination therapy

Hydrogels and nanoparticles are attractive because local delivery could improve lesion-site exposure and reduce systemic toxicity, and both platforms have been reviewed extensively in SCI repair (Silva et al., 2021; Chakraborty et al., 2021). Nevertheless, a lesion containing neurons, oligodendrocytes, astrocytes, microglia, infiltrating macrophages, and vascular cells simultaneously does not become cell-selective merely because delivery is local. Cargo biodistribution and cellular uptake must be measured directly. The specific combination of copper chelation, ncRNA modulation, and epigenetic regulation has not been tested experimentally and should be developed as a staged preclinical program rather than promoted as an established therapeutic package.

Table 3 ranks interventions by evidence level and translational readiness. The table intentionally separates direct SCIRI evidence from traumatic SCI non-cuproptosis evidence and non-SCI delivery or molecular evidence.

**TABLE 3:** Therapeutic strategies targeting the ncRNA–epigenetics–cuproptosis axis.

Strategy	Rationale	Current evidence level	Potential advantage	Main risk or limitation	Development priority
TM or copper chelation	Reduce copper-dependent DLAT aggregation	SCIRI efficacy: TM/ATTM rescue in Xie et al. (2025). Safety/monitoring context only: Wilson disease literature (Brewer, 2009; European Association for the Study of the Liver, 2012).	Mechanistically aligned with cuproptosis	Copper deficiency, uncertain traumatic SCI timing	Validate in traumatic SCI time-course and dose-window studies
D-penicillamine or trientine	Alternative copper-lowering agents	Clinical use in Wilson disease, not SCI (European Association for the Study of the Liver, 2012)	Repurposing feasibility	Slow kinetics and systemic toxicity may not fit acute SCI	Only after biomarker-defined copper dysregulation is shown
miRNA/ASO/siRNA	Cell-type-specific CRG modulation	Non-SCI RNA-therapy experience (Rupaimoole and Slack, 2017)	Programmable target selection	Delivery and off-target effects	Use first as causal validation tools
CRISPR-Cas13	RNA knockdown without DNA editing	Platform-level evidence outside SCI (Abudayyeh et al., 2017)	Reversible RNA targeting	Delivery, immunogenicity, editing specificity	Long-term exploratory
HDAC/BET/DNMT modulation	Shift chromatin states and inflammatory programs	SCI valproic-acid benefit is preclinical and not HDAC-specific (Lv et al., 2012); broader chromatin plausibility exists, but no direct cuproptosis proof is available.	May address multiple secondary-injury modules	Broad effects and cell-type ambiguity	Test CRG-specific chromatin endpoints
Hydrogel or nanoparticle delivery	Local sustained delivery at lesion site	SCI delivery evidence, not cuproptosis-specific (Silva et al., 2021; Chakraborty et al., 2021)	Improved local exposure	Biomaterial inflammation, manufacturing complexity	Pair with validated cargo only

## Biomarkers, clinical diagnosis, and real-world evidence

A clinically useful biomarker program would need to move beyond CRG scores. Serum or CSF copper, ceruloplasmin, non-ceruloplasmin-bound copper, and urinary copper monitoring are established in Wilson disease contexts, but their analytical and clinical validity in acute SCI is unknown (European Association for the Study of the Liver, 2012; Walshe, 2010).

Circulating miRNAs have been explored as severity biomarkers in acute SCI, and MRI-based metrics are among the most mature imaging biomarkers for traumatic SCI prognosis (Hachisuka et al., 2014; Freund et al., 2019). The unresolved question is whether adding copper indices or ncRNA panels improves discrimination beyond American Spinal Injury Association Impairment Scale (AIS) grade, magnetic resonance imaging (MRI) features, timing of decompression, and systemic injury variables.

Prospective human biomarker studies should harmonize neurological classification, injury timing, imaging, biospecimen collection, complications, and outcomes with the ISCoS International SCI Data Sets and the NINDS SCI Common Data Elements to improve cross-cohort comparability (Biering-Sørensen et al., 2012, 2015).

Real-world evidence has not yet tested copper-targeted or cuproptosis-targeted SCI therapy. Registries and pragmatic cohorts are more likely to be useful after a mechanistic biomarker identifies a biologically enriched subgroup. Otherwise, trials risk enrolling heterogeneous patients in whom copper dyshomeostasis is not a dominant injury driver.

## Methodological roadmap

Future SCI-cuproptosis studies should be designed both to test and, if appropriate, to falsify the hypothesis. A minimal traumatic SCI validation package should include contusion or compression models, direct copper mapping by ICP-MS or comparable metallomic imaging, cell-type-resolved CRG expression, lipoylated DLAT/DLST aggregation assays, Fe-S cluster protein measurements, and rescue by both copper chelation and genetic manipulation.

Single-cell and spatial multi-omics can identify candidate cell states, but they should be paired with metallomics and protein assays because mRNA abundance alone cannot define a metal-dependent death pathway (Vandereyken et al., 2023). Large-animal models and human surgical or autopsy samples will be important for translation because rodent lesions differ from human SCI in size, vascular architecture, immune kinetics, and treatment timing (Nardone et al., 2017).

Finally, preregistration, blinded outcome assessment, sex-balanced designs, standardized time points, and public deposition of raw metallomic, imaging, transcriptomic, proteomic, and analysis-code outputs should be treated as translational infrastructure rather than optional extras. DOI-issuing repositories such as Zenodo, Figshare, or Dryad can support persistent access, and metadata should follow FAIR principles of findability, accessibility, interoperability, and reusability (Nosek et al., 2018; Wilkinson et al., 2016).

## Discussion and future priorities for molecular SCI research

The strongest conclusion is that cuproptosis is a real and mechanistically distinct copper-dependent mitochondrial death program and that SCIRI provides the strongest direct preclinical mechanistic spinal-cord evidence to date. The central unresolved issue is whether the same ATP7B–FDX1–DLAT/DLST axis drives clinically relevant tissue loss in traumatic SCI. Methodologically, this targeted narrative review relied on iterative searching and collaborative source appraisal rather than a prospectively deduplicated screening workflow; consequently, database-level retrieval, exclusion, and flow counts cannot be reconstructed, and the descriptive evidence hierarchy should not be interpreted as a pooled risk-of-bias assessment.

The area most likely to be overestimated is immediate therapeutic translation from CRG scores or cross-disease ncRNA networks. The area most likely to be underestimated is methodological integration: copper imaging, spatial transcriptomics, aggregation assays, and cell-type-specific rescue in the same experiment could rapidly clarify whether the field is observing a causal pathway or a stress-associated signature.

**FIGURE 4:**
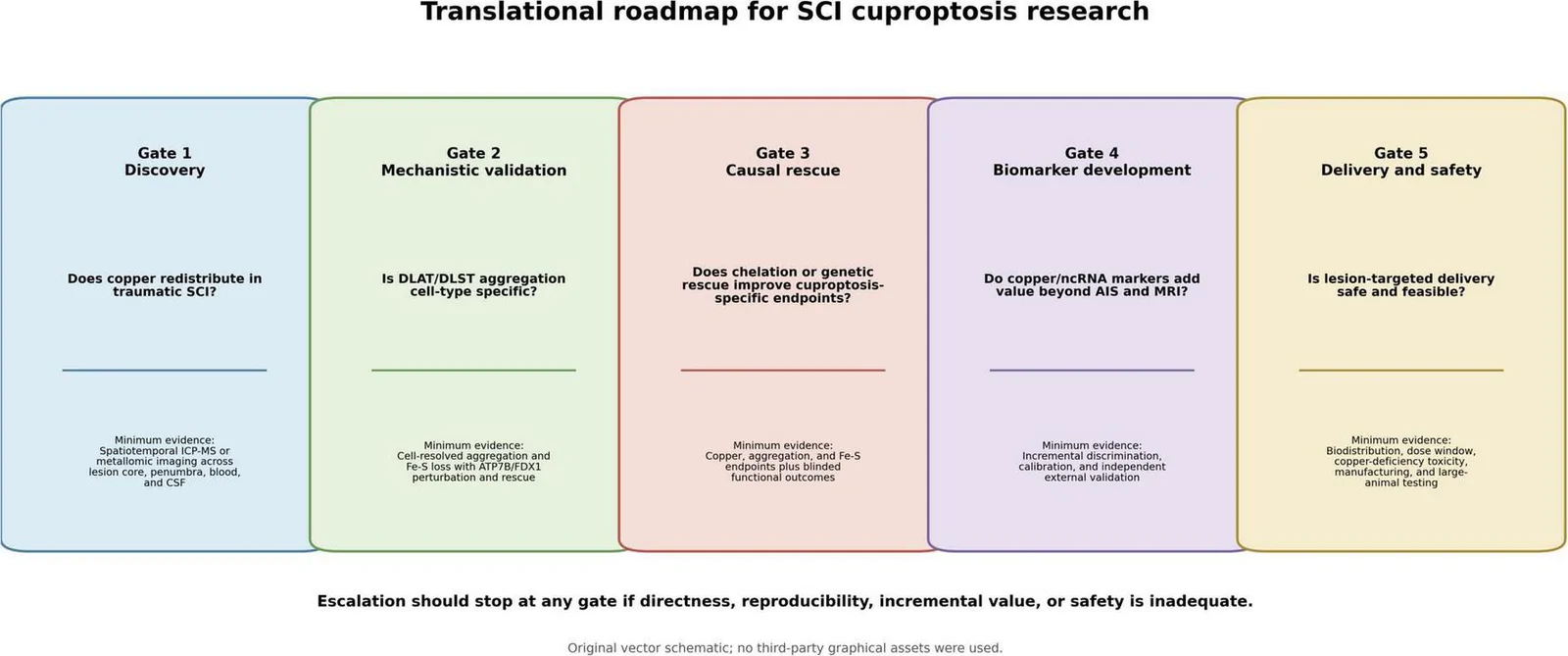
Translational roadmap for SCI cuproptosis research. Five decision gates specify the minimum question before escalation: Gate 1, does copper redistribute in traumatic SCI, demonstrated by spatiotemporal mapping across lesion and systemic compartments? Gate 2, is DLAT/DLST aggregation cell-type specific, supported by spatial and protein-level colocalization? Gate 3, does chelation or genetic rescue improve cuproptosis-specific copper, aggregation, and Fe-S endpoints as well as blinded functional outcomes? Gate 4, do copper/ncRNA biomarkers add value beyond AIS and MRI, shown by incremental discrimination, calibration, and external validation? Gate 5, is lesion-targeted delivery safe and feasible, including biodistribution, dose window, copper-deficiency toxicity, manufacturing, and large-animal assessment? Escalation should stop if a gate is not met. The figure was created with original vector shapes; no third-party graphical assets were used.

We consider the highest-priority translational path to be a biomarker-guided validation pipeline: first define when and where copper redistribution occurs, then identify susceptible cell states, then test whether copper-lowering or RNA/epigenetic modulation rescues cuproptosis-specific endpoints without impairing copper-dependent physiology. This staged roadmap is intended to support molecularly precise SCI research prior to any clinical translation (Figure 4).

## Search strategy and evidence selection

This targeted narrative review used an iterative, question-oriented search rather than a prospectively registered systematic-review workflow. PubMed, Web of Science Core Collection, and Google Scholar were searched from inception to 15 April 2026 using combinations of terms related to SCI/SCIRI, cuproptosis and copper homeostasis, ncRNAs, epigenetic regulation, biomarkers, regulated cell death, and delivery strategies. PubMed was used for bibliographic identification, whereas Web of Science Core Collection and Google Scholar supported citation tracing and discoverability. Reference lists of key mechanistic and SCI-focused articles were also examined. Search outputs and key sources were reviewed collaboratively within the author team during manuscript development. This process was not structured as formal independent duplicate screening; differences in evidence interpretation were resolved through discussion. Sources were prioritized according to their relevance to the review questions and their contribution to a predefined descriptive hierarchy: established cuproptosis biochemistry; the strongest direct preclinical mechanistic evidence in spinal-cord models; traumatic-SCI associative or bioinformatic evidence; non-SCI mechanistic evidence; and testable hypotheses. Because the searches were iterative and were not managed as a single prospectively deduplicated screening set, database-level retrieval counts, duplicate-removal counts, stage-specific exclusion counts, and a numerical flow diagram were not retrospectively reconstructed. Database-specific search syntax, evidence-selection principles, and the methodological limitation are provided in Supplementary material. Several references published or indexed after 15 April 2026 were added during peer-review revision for citation verification and to maintain currency; these targeted additions were not treated as part of the original iterative search set.
