# Supplementary material for: Copper-regulated cell death after spinal cord injury: evidence boundaries for non-coding RNA, epigenetics and cuproptosis
Source: Front Mol Neurosci. 2026 Sep 10;19:1885261. doi: 10.3389/fnmol.2026.1885261 (PMC13601787; doi:10.3389/fnmol.2026.1885261)
Supplement: Supplementary file 1 [file Data_Sheet_1.pdf]

Supplementary Data Sheet 1. Methods and Evidence Appraisal

Scope note: This supplement documents a targeted narrative-review search framework and evidence-selection process. It is not a PRISMA record of exhaustive study selection and intentionally contains no retrospective retrieval or exclusion counts.

S1. Search framework and evidence-selection principles

Purpose. This supplement documents how literature was identified, prioritized, and interpreted. The search was iterative and question-oriented, supporting a critical narrative synthesis rather than an exhaustive systematic review.

Review process. Search outputs and key sources were reviewed collaboratively within the author team during manuscript development. This process was not structured as formal independent duplicate screening; differences in evidence interpretation were resolved through discussion.

Full-text verification. Publications used to support central mechanistic claims were checked against accessible full text or the publisher record whenever available. Records for which the relevant evidence could not be verified were not used to substantiate central mechanistic conclusions. A separate numerical count of unavailable full texts was not prospectively logged.

Methodological limitation. Because the searches were iterative and were not managed as a single prospectively deduplicated screening set, database-level retrieval counts, duplicate-removal counts, stage-specific exclusion counts, and a numerical flow diagram cannot be reconstructed reliably. The review therefore does not claim exhaustive coverage or provide quantitative estimates of search completeness. Its purpose is to map evidence boundaries and experimentally resolvable gaps.

S2. Database-specific search syntax

Supplementary Table S1. Database-specific search syntax and search role.

Module	Source	Search syntax or role
A. Direct SCI/SCIRI copper–cuproptosis evidence	PubMed	(("Spinal Cord Injuries"[Mesh] OR "spinal cord injury"[Title/Abstract] OR "traumatic spinal cord injury"[Title/Abstract] OR "spinal cord ischemia reperfusion injury"[Title/Abstract] OR "spinal cord ischemia-reperfusion injury"[Title/Abstract] OR SCIRI[Title/Abstract]) AND (cuproptosis[Title/Abstract] OR "copper-induced cell death"[Title/Abstract] OR "copper-regulated cell death"[Title/Abstract] OR "copper homeostasis"[Title/Abstract] OR FDX1[Title/Abstract] OR DLAT[Title/Abstract] OR DLST[Title/Abstract] OR ATP7A[Title/Abstract] OR ATP7B[Title/Abstract] OR SLC31A1[Title/Abstract])) AND ("1900/01/01"[Date - Publication] : "2026/04/15"[Date - Publication])
A. Direct SCI/SCIRI copper–cuproptosis evidence	Web of Science Core Collection	TS=(("spinal cord injury" OR "traumatic spinal cord injury" OR "spinal cord ischemia reperfusion injury" OR "spinal cord ischemia-reperfusion injury" OR SCIRI) AND (cuproptosis OR "copper-induced cell death" OR "copper-regulated cell death" OR "copper homeostasis" OR FDX1 OR DLAT OR DLST OR ATP7A OR ATP7B OR SLC31A1)) AND PY=(1900-2026)
A. Direct SCI/SCIRI copper–cuproptosis evidence	Google Scholar	Targeted combinations such as: "spinal cord injury" cuproptosis copper FDX1 DLAT ATP7B;

Module	Source	Search syntax or role
		used iteratively for discoverability and backward/forward citation tracing.
B. SCI ncRNA and epigenetic context	PubMed	(("Spinal Cord Injuries"[Mesh] OR "spinal cord injury"[Title/Abstract] OR "traumatic spinal cord injury"[Title/Abstract] OR "spinal cord ischemia reperfusion injury"[Title/Abstract]) AND ("non-coding RNA"[Title/Abstract] OR "noncoding RNA"[Title/Abstract] OR miRNA[Title/Abstract] OR microRNA*[Title/Abstract] OR lncRNA*[Title/Abstract] OR circRNA*[Title/Abstract] OR "DNA methylation"[Title/Abstract] OR "histone modification*"[Title/Abstract] OR epigenetic*[Title/Abstract] OR m6A[Title/Abstract] OR "N6-methyladenosine"[Title/Abstract] OR m5C[Title/Abstract])) AND ("1900/01/01"[Date - Publication] : "2026/04/15"[Date - Publication])
B. SCI ncRNA and epigenetic context	Web of Science Core Collection	TS=(("spinal cord injury" OR "traumatic spinal cord injury" OR "spinal cord ischemia reperfusion injury") AND ("non-coding RNA" OR "noncoding RNA" OR miRNA OR microRNA* OR lncRNA* OR circRNA* OR "DNA methylation" OR "histone modification*" OR epigenetic* OR m6A OR "N6-methyladenosine" OR m5C)) AND PY=(1900-2026)
C. Non-SCI ncRNA/epigenetic regulation of cuproptosis	PubMed	((cuproptosis[Title/Abstract] OR "copper-induced cell death"[Title/Abstract] OR "copper-regulated cell death"[Title/Abstract]) AND ("non-coding RNA"[Title/Abstract] OR "noncoding RNA"[Title/Abstract] OR miRNA[Title/Abstract] OR microRNA*[Title/Abstract] OR lncRNA*[Title/Abstract] OR circRNA*[Title/Abstract] OR "DNA methylation"[Title/Abstract] OR "histone modification*"[Title/Abstract] OR epigenetic*[Title/Abstract] OR m6A[Title/Abstract] OR m5C[Title/Abstract])) AND ("1900/01/01"[Date - Publication] : "2026/04/15"[Date - Publication])
C. Non-SCI ncRNA/epigenetic regulation of cuproptosis	Web of Science Core Collection	TS=((cuproptosis OR "copper-induced cell death" OR "copper-regulated cell death") AND ("non-coding RNA" OR "noncoding RNA" OR miRNA OR microRNA* OR lncRNA* OR circRNA* OR "DNA methylation" OR "histone modification*" OR epigenetic* OR m6A OR m5C)) AND PY=(1900-2026)
D. Regulated-cell-death crosstalk in traumatic SCI	PubMed	(("traumatic spinal cord injury"[Title/Abstract] OR "spinal cord injury"[Title/Abstract]) AND (ferroptosis[Title/Abstract] OR pyroptosis[Title/Abstract] OR necroptosis[Title/Abstract] OR autophagy[Title/Abstract] OR mitophagy[Title/Abstract])) AND ("1900/01/01"[Date - Publication] : "2026/04/15"[Date - Publication])

S3. Descriptive evidence hierarchy

Supplementary Table S2. Descriptive evidence hierarchy used in the review.

Level	Label	Minimum supporting features	Inference boundary
Level 1	Established canonical biochemistry	Direct mechanistic evidence for	Does not establish occurrence in

Level	Label	Minimum supporting features	Inference boundary
		copper binding to lipoylated mitochondrial proteins, aggregation, Fe-S protein loss, and pathway-specific perturbation.	SCI.
Level 2	Strongest direct preclinical mechanistic evidence in spinal-cord models	SCIRI or traumatic-SCI study with direct copper measurement plus execution markers and perturbation/rescue.	Model-specific; SCIRI is not equivalent to traumatic SCI or human evidence.
Level 3	Associative/hypothesis-generating traumatic-SCI evidence	Bulk/single-cell expression, CRG scores, immune associations, machine learning, or candidate biomarkers.	Cannot establish cuproptosis execution without copper, protein aggregation, and rescue.
Level 4	Non-SCI mechanistic validation	Direct ncRNA or epigenetic regulation of cuproptosis outside SCI.	Supports plausibility only until replicated in SCI-relevant cells/tissue.
Level 5	Testable hypothesis	Mechanistically coherent proposal lacking direct validation.	Must be labeled explicitly and not used to imply therapeutic readiness.

S4. SANRA self-appraisal

This is an author self-appraisal using SANRA and should not be interpreted as independent quality certification.

Supplementary Table S3. SANRA self-appraisal of the revised narrative review.

SANRA item	Score (0–2)	Rationale
Justification of importance	2	The review identifies the secondary-injury problem and the risk of overinterpreting CRG signatures.
Statement of aims	2	A central causal and translational question is stated explicitly.
Description of literature search	2	Databases, date, exact syntax, citation tracing, selection principles, collaborative review process, and limitations are reported.
Referencing	1	Central claims are referenced and named recent records were verified; the review is not exhaustive.
Scientific reasoning	2	Direct, associative, extrapolated, and hypothetical evidence are separated throughout.
Presentation of relevant data	1	Key mechanistic and study-design features are summarized, but no quantitative pooling was intended.

Total self-appraisal score: 10/12. The score reflects the revised manuscript and this supplement.

S5. Reporting and validation appraisal of anchor evidence

The table assesses reporting completeness and validation depth, not a formal pooled risk-of-bias judgment. “Not clearly reported” means that the feature was not identifiable in the report reviewed; it does not prove that the procedure was not performed.

Supplementary Table S4. Reporting and validation appraisal of anchor and representative evidence.

Study/design	Sample/design information	Randomization, blinding, and calculation	Validation depth	Interpretive limit
Xie et al., 2025; rat SCIRI plus OGD/R neuronal models	Transcriptomics and several tissue/cell endpoints used small endpoint-specific samples (commonly n=3); locomotor assessment n=6/group.	Group randomization and a priori sample-size calculation were not clearly reported. Locomotor scoring was performed by two investigators blinded to group allocation.	Direct copper accumulation, ATP7B/FDX1 changes, lipoylated DLAT aggregation, Fe-S loss, and TM/ATTM rescue.	Strongest direct preclinical mechanistic spinal-cord evidence, but limited by one male SCIRI model, small endpoint-specific n, and incomplete reporting of allocation/concealment.

Study/design	Sample/design information	Randomization, blinding, and calculation	Validation depth	Interpretive limit
Li et al., 2023; DLD-centered acute-SCI bioinformatics	GSE151371 peripheral-blood leukocyte expression data; no lesion-tissue copper or aggregation assay.	Randomization/blinding not applicable to public-data analysis; analytic overfitting and cell-composition confounding remain relevant.	Association with immune microenvironment; no independent causal or copper-specific rescue.	Level 3 associative/hypothesis-generating evidence.
Mao et al., 2025; integrated bioinformatics	Multiple bulk RNA-seq time points, an external single-cell cohort, and in-vitro validation.	Randomization/blinding not applicable to the primary computational analyses; experimental reporting should be interpreted separately.	External single-cell corroboration and in-vitro functional work improve within-tier credibility.	Stronger Level 3 evidence than an unvalidated signature, but Mpeg1 remains a hub association rather than direct cuproptosis proof.
Zhou et al., 2025; machine-learning model plus rat validation	GSE151371 human peripheral-blood dataset (38 SCI and 10 controls reported in the source study) plus rat behavioral, histological, ultrastructural, and qRT-PCR experiments.	Model development and evaluation were based mainly on the same human dataset; independent human external validation was limited. Animal randomization/blinding and sample-size calculation should be checked in the source report.	Rat experiments corroborated selected genes but did not provide a complete copper–aggregation–rescue chain.	Level 3 associative evidence with experimental corroboration, not causal proof.
Li et al., 2018; miR-133b-modified MSC exosomes in SCI	Preclinical SCI exosome study supporting EV-miRNA delivery and recovery.	Study-level randomization/blinding should be interpreted from the source report; not appraised here as cuproptosis evidence.	Supports EV-miRNA benefit in SCI but includes no CRG/copper/cuproptosis endpoint.	Relevant to delivery plausibility only; it does not validate ncRNA regulation of cuproptosis.

S6. Verification of reviewer-queried recent references

Supplementary Table S5. Verification of recent references queried during peer review.

Record	Verified citation	DOI	Audit note
Hsiao et al.	Nat Commun. 2026;17:1112.	10.1038/s41467-025-67869-0	Publisher citation verified; online publication and volume year differ because the DOI was registered in 2025.
Li et al.	Ann Med. 2026;58:2641867.	10.1080/07853890.2026.2641867	Verified.
Ni et al.	Neural Regen Res. 2026;21(7):2824–2842.	10.4103/NRR.NRR-D-24-01449	Verified.
Du et al.	Mol Cancer. 2025;24:230.	10.1186/s12943-025-02434-3	Verified.
El-Ashmawy et al.	Arch Biochem Biophys. 2025;765:110324.	10.1016/j.abb.2025.110324	Verified.

S7. Data standards and FAIR-oriented roadmap

Prospective human biomarker studies should align neurological classification, injury timing, imaging, biospecimen, complication, and outcome variables with the ISCoS International SCI Data Sets and NINDS SCI Common Data Elements. Raw metallomic, imaging, transcriptomic, proteomic, and code outputs should be deposited in repositories that issue persistent identifiers, with metadata and reusable workflows consistent with FAIR principles.

Supplementary references

Baethge, C., Goldbeck-Wood, S., Mertens, S. (2019). SANRA—a scale for the quality assessment of narrative review articles. Res Integr Peer Rev 4, 5. doi: 10.1186/s41073-019-0064-8

Biering-Sørensen, F., Charlifue, S., DeVivo, M.J., et al. (2012). Using the Spinal Cord Injury Common Data Elements. Top Spinal Cord Inj Rehabil 18(1), 23–27. doi: 10.1310/sci1801-23

Biering-Sørensen, F., Alai, S., Anderson, K., et al. (2015). Common data elements for spinal cord injury clinical research: a National Institute for Neurological Disorders and Stroke project. Spinal Cord 53(4), 265–277. doi: 10.1038/sc.2014.246

Percie du Sert, N., Hurst, V., Ahluwalia, A., et al. (2020). The ARRIVE guidelines 2.0: Updated guidelines for reporting animal research. PLoS Biol 18(7), e3000410. doi: 10.1371/journal.pbio.3000410

Wilkinson, M.D., Dumontier, M., Aalbersberg, I.J.J., et al. (2016). The FAIR Guiding Principles for scientific data management and stewardship. Sci Data 3, 160018. doi: 10.1038/sdata.2016.18
